# Live Imaging of Innate and Adaptive Immune Responses in the Liver

**DOI:** 10.3389/fimmu.2020.564768

**Published:** 2020-09-17

**Authors:** Lu Li, Zhutian Zeng

**Affiliations:** ^1^The First Affiliated Hospital of USTC, Division of Life Sciences and Medicine, University of Science and Technology of China, Hefei, China; ^2^Hefei National Laboratory for Physical Sciences at Microscale, The CAS Key Laboratory of Innate Immunity and Chronic Disease, School of Basic Medical Sciences, Division of Life Sciences and Medicine, University of Science and Technology of China, Hefei, China

**Keywords:** intravital imaging, Kupffer cell, iNKT cell, NK cell, T cells, infection, liver disease

## Abstract

Immune response in the liver is determined by the spatial organization and cellular dynamics of hepatic immune cells. The liver vasculature accommodates abundant tissue-resident innate immune cells, such as Kupffer cells, natural killer cells, and natural killer T cells, to ensure efficient intravascular immunosurveillance. The fenestrated sinusoids also allow direct contact between circulating T cells and non-canonical antigen-presenting cells, such as hepatocytes, to instruct adaptive immune responses. Distinct cellular behaviors are exploited by liver immune cells to exert proper functions. Intravital imaging enables real-time visualization of individual immune cell in living animals, representing a powerful tool in dissecting the spatiotemporal features of intrahepatic immune cells during steady state and liver diseases. This review summarizes current advances in liver immunology prompted by *in vivo* imaging, with a particular focus on liver-resident innate immune cells and hepatic T cells.

## Introduction

Receiving blood from the gastrointestinal tract via the portal vein, the liver stands out as one of the prominent interfaces constitutively exposed to numerous food antigens, environmental toxins, and commensal-derived microbial products. This unique anatomy of the liver profoundly shapes its immunological properties (1, 2). In the liver, the sinusoidal blood is actively scanned by a dense network of intravascular macrophages, namely, Kupffer cells (KCs), which represent the largest population of tissue macrophages in our body. The liver is also enriched in many other innate immune cells, including natural killer T (NKT) cells, natural killer (NK) cells, and γδ-T cells; they are fully equipped immune effectors with potential to patrol around the tissue. The predominance of innate immune cells endows the liver with an ability to rapidly combat foreign invaders (3). By contrast, to minimize the unwanted immune response against harmless antigenic stimuli, e.g., food antigens and metabolic by-products, adaptive immunity in the liver is usually blunted due to the immunotolerogenic liver-resident antigen-presenting cells (APCs) (4, 5). With these distinct immunological features, the liver has long been considered as an immune organ (6, 7). It was proposed as “an immune barrier” (8), “an organ with predominant innate immunity” (3), “a school to educate regulatory immune cells” (9), and “a graveyard for T cells” (10), each points out a specific function of immune cells in maintaining liver homeostasis or in regulating systemic immune responses. Intravital microscopy (IVM) has aided in deciphering the function of liver immune cells at steady state and disease and, therefore, has greatly improved our understanding of liver immunology.

The most widely used techniques for immunological researches, such as multiplex flow cytometry and immunofluorescence, have provided fruitful information into the composition, abundance, and phenotype of hepatic immune cells. Recent advances in single-cell sequencing have further uncovered the heterogeneity of liver immune cells via unbiased transcriptomic analysis (11, 12). However, these *ex vivo* approaches inevitably rely on cell isolation or tissue slicing, during which immune cells undergo enzymatic digestion, vortex, or fixation. These procedures may have impacts on the viability, phenotype, activation status, and even function of immune cells (13). Most importantly, immune cells are highly diverse in terms of their motility, behavior, and cellular interaction; all of these properties are of important relevance to immune cell functions but are usually neglected by the aforementioned *ex vivo* cell profiling methods.

IVM is a state-of-the-art technique to visualize cells over time in living animals through a high-resolution fluorescence confocal microscope. It enables a single-cell level tracking of individual cells *in situ* and in real time, without the need to isolate the cells. Therefore, IVM becomes a versatile and powerful tool in many fields of biomedical researches, such as immunology, tumor biology, and cell biology (14–16). Intravital imaging of immune cell dynamics in the mouse liver can be readily performed by externalization of one liver lobe or by implantation of an optical abdominal window. Under a spinning disk or laser scanning confocal microscope, a variety of hepatic immune cell populations have been visualized by utilizing different fluorescent reporter mouse strains or dyes (Table 1) (17). The cellular dynamics of these cells have been recorded in physiological or pathological conditions, bringing new perspectives into the function of liver immune cells. In this review, we will describe how IVM advances our understanding of liver immunology, with a focus on liver-resident innate immune cells and hepatic T cell responses.

**TABLE 1:** Strategies for visualizing hepatic immune cell population by IVM.

Cell types	Surface markers	Labeling methods for IVM
Kupffer cell	CD11b^lo^F4/80^hi^Tim4^+^Clec4f^+^	Dye-conjugated anti-F4/80 or anti-TIM-4 antibody*
iNKT cell	CD3^+^NK1.1^+^α-Galcer/CD1d tetramer^+^	CXCR6-GFP reporter
LrNK cell	CD3^–^DX5^–^NK1.1^+^CD49a^+^	Not reported
cNK cell	CD3^–^DX5^+^NK1.1^+^CD49a^–^	Ncr1-cre^†^ × tdTomato reporter^‡^
CD8^+^ T cell	CD3^+^NK1.1^–^CD8^+^	dye-conjugated anti-CD8 antibody, transfer of pre-labeled T cells;
CD4^+^ T cell	CD3^+^ NK1.1^–^CD4^+^	Dye-conjugated anti-CD4 antibody; transfer of prelabeled T cells
Neutrophil	CD11b^+^Ly6G^+^	Lysm-GFP reporter^§^, Ly6G-cre × tdTomato reporter, dye-conjugated anti-Ly6G antibody
Monocyte	CD11b^+^Ly6C^hi^ or CD11b^+^Ly6C^lo^	CCR2-RFP or CX_3_CR1-GFP reporter^——^

*For labeling cells *in vivo* using dye-conjugated antibodies, a total of 1–2 μg antibodies were injected intravenously into mouse about 10 min before imaging. ^†^Ncr1: natural cytotoxicity triggering receptor 1, specifically expressed on NK cells. ^‡^tdTomato reporter: Rosa26-Loxp-Stop-Loxp-tdTomato mouse. Cre-mediated cleavage of Stop causes tdTomato expression in Cre-expressing cells. This reporter strain can be replaced by Rosa26-LSL-ZsGreen reporter as well. ^§^ GFP^*hi*^ cells in this reporter are almost exclusively neutrophils; GFP^*lo*^ cells contain macrophages and monocytes. ^——^GFP cells in the subcapsular space of liver are capsular macrophages. The transition of monocytes can be imaged by using CCR2-RFP/CX_3_CR1-GFP double reporters.

## Intravital Imaging Provides Spatiotemporal Insights Into Liver-Resident Innate Immune Cells

Tissue-resident immune cells are essential for maintaining tissue integrity during homeostasis and perturbations (18). Defining a tissue-resident immune cell subset has been most commonly achieved by performing parabiosis to check if these cells recirculate or not. This method, however, is time consuming. Alternatively, tissue residency of immune cells can be validated via IVM, which offers essential information on the spatial organization and dynamic behaviors of tissue-resident immune cells. With this cutting-edge technique, the liver-resident innate immune cells, especially macrophages and iNKT cells, have been extensively studied, shedding new light on their function in liver inflammation and infection.

### Kupffer Cells Are Immune Sentinels in the Liver Sinusoids With Blood-Filtering Function

**FIGURE 1:**
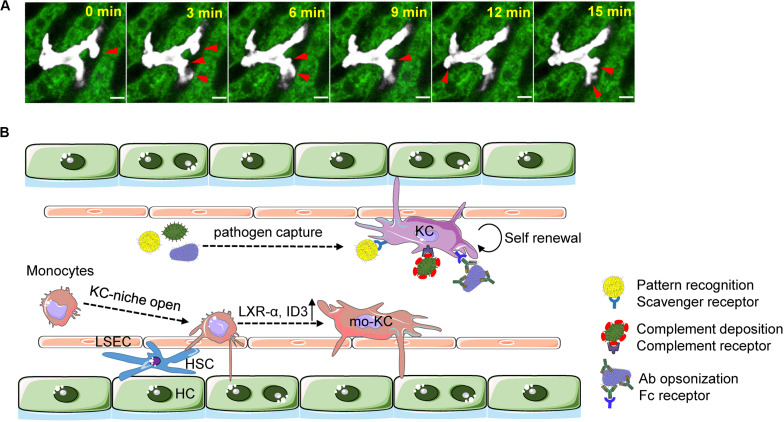
Kupffer cell as immune sentinels with blood-filtering function. **(A)** Time-lapse intravital imaging of a single KC (white pseudocolor) showing the “sampling” behavior of KCs. Red arrows indicate the extending of cell protrusions. Hepatocytes are shown by green autofluorescence. Liver sinusoids are seen as black space between hepatocytes. Scale, 10 μm. **(B)** KCs residing inside the liver vasculature are self-maintained during homeostasis. They capture and phagocytose blood-borne pathogens (bacteria, virus, parasite, fungi, etc.) via various molecular mechanisms involving scavenger receptor-mediated pattern recognition and complement or antibody-dependent opsonization. The “open status” of KC niche as a consequence of KC loss results in rapid infiltration of monocytes. The latter cells adopt a monocyte-derived KC identity by upregulating of transcriptional factors liver X receptor (LXR)-α and inhibitor of DNA binding 3 (ID3). Cellular interactions with hepatocytes, HSCs, and LSECs orchestrate the differentiation of monocytes into KCs.

KCs comprise 80–90% of all body macrophages (19). These very abundant, large, and ramified macrophages fill the thin liver capillaries, constituting a tremendous intravascular phagocyte network that ensures efficient immune surveillance over the liver sinusoidal blood. KCs are not exclusively inside the vasculature; a substantial fraction of their cell body were seen in the perivascular space interacting with hepatic stellate cells (HSCs) and hepatocytes (20). Concordantly, KCs frequently extend cell protrusions, forming a lamellipodium-like structure that is continually scanning back and forth (Figure 1). This prototypical “sampling” behavior might be a reflection of micropinocytosis (21), which may occur actively for KCs to engulf toxic solute in the blood and the space of Disse.

KCs possess an incredible capacity to rapidly clear blood-borne pathogens. Real-time imaging showed that KCs captured and subsequently internalized 80% of inoculated bacteria in <2 min, pinpointing a pivotal function of KCs in preventing systemic bacterial dissemination (22). Complement receptor of the Ig superfamily (CRIg), a well-known receptor for the complement component C3b and iC3b (23), was critical in this biological process. In addition to catching C3b- and iC3b-coated bacteria (23, 24), CRIg was able to directly recognize and bind lipoteichoic acid, a pathogen-associated molecular pattern that is widely expressed on the surface of Gram-positive bacteria (22). This pattern recognition role of CRIg enables almost instant sequestration of Gram-positive bacteria from blood without the need for complement activation and opsonization (22), thereby maximizing the ability of KCs in preventing early bacteria dissemination. CRIg expression was largely restricted to tissue macrophages, particular in KCs (25, 26). This expression profile correlates with the indispensable role of CRIg in KC-mediated immune clearance of various blood-borne microbial species, including parasites (27), fungi (28), and virus (29), all in a complement-dependent manner.

It is believed that KC employs multiple mechanisms other than CRIg to sequester circulating pathogens. Time-lapse analysis of bacterial trapping in the liver revealed a sex-biased difference during enteropathogenic *Escherichia coli* (EPEC) infection. Females possessed an abundant level of estrogen-elicited natural antibodies against EPEC, rendering a faster bacterial capture by KCs than that in males (30). immunoglobulin M (IgM) antibodies turned out to be highly efficient in supporting bacterial capture, although the corresponding receptors remain yet to be defined (30).

KCs work cooperatively with liver sinusoidal endothelial cells (LSECs) to efficiently remove particles in circulation, ranging in size from nanometer to micrometer. This scavenger function of liver dramatically hinders the delivery of therapeutic nanoparticles into target tissues. Up to 99% of systemically administered nanoparticles were trapped and cleared within the liver, mainly by KCs and LSECs (31). Whereas small nanoparticles were uptaken by both cell types, large nanoparticles were cleared preferentially by KCs (31, 32). Similarly, latex beads larger than 1 μm were captured predominantly by KCs (33). The KC’s propensity to phagocytose large particles also held true for immune complexes (ICs); large ICs generated by a bispecific antibody were almost exclusively uptaken by KC, but small ICs preferentially ended up in LSECs (34).

Efferocytosis of unhealthy blood cells emerges as another important blood filter function of KCs. The liver, in addition to the spleen and bone marrow, has been proposed as a major place to clear aged blood cells (35). However, definitive evidence by real-time visualization of blood cell clearance in the liver is scarce. Recent imaging-based studies demonstrated that aged or injured platelets were trapped and removed by KC but neither hepatocytes nor LSECs. This function of KCs relied on a collaboration of their macrophage galactose lectin and Ashwell–Morell receptor to capture desialylated platelets from the blood (36). Whereas C-type lectin domain family 4 member f (CLEC4f) as the KC specific receptor was proposed to capture desialylated platelets in mouse (37), this receptor was absent in human (38). Transformed cells, including metastatic cancer cells, can be trapped in the liver at least partly by KCs, which expressed a full array of scavenger receptors and lectin receptors that elegantly discriminated the “eat me” and “don’t eat me” signals exposed on tumor cells (39, 40). Antibody opsonization potentiated KC-mediated elimination of circulating tumor cells, as seen *in vivo* during antibody treatment of B cell lymphoma, melanoma cells, and colon carcinoma cells (41, 42).

### Imaging the Heterogeneity and Replenishment of Liver Macrophages

The ontogeny and diversity of liver macrophages have recently drawn substantial attention. KCs originated from embryonic precursors and are self-maintained. Distinct subsets of KCs at steady state has been observed based on the differences in cell size (43, 44), localization (43, 44), surface marker (43, 45), and transcriptome (12, 46). Monocytes also contribute to liver macrophage pool upon KC loss during infection or injury (47, 48). Although these monocyte-derived macrophages appear as transient infiltrating cells that exert on-demand proinflammatory, reparative, or erythrophagocytic functions (49–51), some of them may establish long-term tissue residency with time especially when the KC niche is wide open (52). This results in a population of liver-resident macrophages that can be functionally and phenotypically different from embryonically derived KCs (48, 53–55). Understanding the heterogeneity of liver macrophages may have important implications for treating liver diseases (56).

A recent imaging-based study elaborately illustrated the cellular interactions required for monocyte-mediated macrophage replenishment in the liver (20). Monocytes rapidly infiltrated the liver upon KC depletion, increased cell size, and adopted an elongated shape with big processes, resembling a prototypical KC morphology. Their pseudopods protruded through LSECs to interact with HSCs in the space of Disse and concurrently to reach hepatocytes. These cell–cell interactions collectively imprinted a KC identity on differentiating monocytes by providing essential molecular cues to drive KC development (20, 57). Therefore, the three major hepatic cell types, including LSECs, HSCs, and hepatocytes, composed a liver-specific macrophage niche to orchestrate the differentiation of precursors into KCs.

Intravital imaging helped discover a new liver macrophage population located right below the liver capsule (55, 58), a region that was overlooked by the liver immunological studies in the past. These capsular cells were found to inhabit the extravascular space under a thin layer of liver mesothelium and were identified by their uniform expression of C-X3-C motif chemokine receptor 1 (CX_3_CR1), a marker that was absent on KCs. They were first reported as liver dendritic cells (DCs) (55) but were later recognized as macrophages because a panel of key macrophage markers were detected, including F4/80, CD64, CD14, and colony stimulating factor 1 receptor (CSF1-R) (58). Antibody staining of these capsular cells *in vivo* took much longer than expected, which could be the major reason causing the discrepancy between these two studies in classifying these cells. CX_3_CR1^+^ capsular cells exhibited a “sampling” behavior and were able to sense and catch bacteria that breached the liver mesothelium from peritoneum (58), suggesting their potential to act as immune sentinels in the subcapsular liver area. The functional importance of capsular macrophages in liver diseases remains to be uncovered; their crosstalk with intravascular immune cells or other subcapsular cell populations merits further investigation.

### Liver Resident iNKT Cells Patrol the Liver Sinusoids

Invariant NKT (iNKT) cells can acquire long-term tissue residency in a non-lymphoid organ (18, 59), particularly in the liver. Up to 30% of total intrahepatic lymphocytes in mice and 10% of that in human are iNKT cells (3). A C-X-C motif chemokine receptor 6-green fluorescent protein C-X-C motif chemokine receptor 6–green fluorescent protein (CXCR6-GFP) reporter mouse strain has been widely used to image iNKT cells *in vivo*. More than three quarters of GFP^hi^ cells in the liver of these mice are CD1d-restricted iNKT cells (60). Hepatic iNKT cells were seen to slowly crawl along the liver sinusoids without a directional bias, representing an intravascular patrolling behavior that was distinguishable from leukocyte rolling along vascular endothelium (33, 60). The molecular basis underlying iNKT cell patrolling remains unclear. CXCR6 and its ligand C-X-C motif chemokine ligand 16 (CXCL16) played no role despite their abundant and constitutive expression on iNKT cells and liver endothelium respectively (60). Instead, this chemokine signaling functioned to maintain the survival of hepatic iNKT cells (60) and to attract circulating iNKT cells into the inflamed liver during injury (61) and cancer (62). Recent findings suggested an essential role of integrin-mediated cell adhesion for iNKT cells to retain in liver sinusoids. Blockade of lymphocyte function-associated antigen 1 (LFA-1) and its ligand intercellular adhesion molecule 1 (ICAM-1) in tandem abolished hepatic retention of iNKT cells, causing their redistribution from the liver into blood (63). Transcriptional factors, including promyelocytic leukemia zinc finger (PLZF) (63), B-lymphocyte-induced maturation protein 1 (Blimp1), and homolog of Blimp1 in T cells (Hobit) (64) were indispensable for the tissue residency of iNKT cells, possibly by upregulating integrins and by suppressing lymphocyte egress genes.

Patrolling iNKT cells underwent rapid change of cellular behavior upon encountering cognate antigens; they stopped crawling and became stationary within 1 h after iNKT cell agonist α-galactosylceramide (α-Galcer) administration (60). A similar response was induced by inflammatory cytokines, indicating that the arrest of iNKT cell movement was a general result of cell activation. This was confirmed by the upregulation of CD69 and the production of effector cytokines by arrested iNKT cells (65).

**FIGURE 2:**
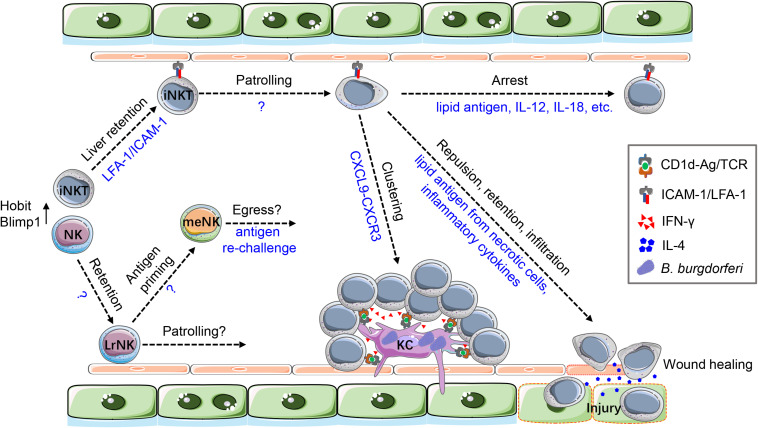
The cellular dynamics of iNKT and NK cells in the liver. A large number of iNKT and NK cells retain in the liver under the control of Hobit and Blimp1. LFA-1/ICAM-1 interaction is critical for iNKT cells to retain in the liver. Hepatic iNKT cells show an intravascular patrolling behavior along the endothelium, but the underlying molecular mechanisms are unknown. Patrolling iNKT cells quickly arrest their movement and become activated in response to cognate antigens and innate cytokine (IL-12, IL-18, etc.). During blood-borne *B. burgdorferi* infection, KCs capture the bacteria and attract iNKT cell via CXCL9-CXCR3 chemokine signaling. iNKT cells then form clusters surrounding KCs and get activated by CD1d-presented lipid antigens. In turn, activated iNKT cells prevent bacteria from escaping possibly by enhancing bacterial killing via IFN-γ production. In liver sterile injury, endogenous lipid antigens from necrotic hepatocytes and inflammatory cytokines from macrophages orchestrate a multistep response of hepatic iNKT cells, including repulsion, retention, and infiltration. iNKT cells are activated during this process and produce IL-4 to promote wound healing at the injured site. The spatiotemporal features of LrNK cells are much less understood. It remains unclear how LrNK cells are retained in the liver and whether LrNK cells patrol in the sinusolids. Moreover, during LrNK cell-mediated memory response, the cellular dynamics of LrNK cell priming and egress merit further investigation.

Characterizing the spatiotemporal features of iNKT cells has provided novel insights into the activation and function of these cells (Figure 2). During bloodstream *Borrelia burgdorferi* infection, iNKT cells gradually decreased their crawling velocity and became completely immotile by 24 h postinfection. Arrested iNKT cells were closely abutted to bacteria-containing KCs and formed stable clusters. This iNKT cell clustering maybe a strategy to enhance the killing of intracellular bacteria via augmenting the local concentration of interferon-γ (IFN-γ), which was released by activated iNKT cells (33). The response of iNKT cells to sterile injury can be categorized into three phases in a well-studied focal liver burn injury model (66). In the early repulsion phase, patrolling iNKT cells approached the lesion but made a U-turn at the border and then retreated. In the mid-retention phase, iNKT cells were arrested and accumulated as a ring structure around the injured area. In the late infiltration phase, iNKT cells finally migrated across the boundary and infiltrated the injury site, where they promoted wound healing by producing interleukin-4 (IL-4) (67). Local signals, including endogenous glycolipids from necrotic cells and the inflammatory cytokines produced by KCs, coordinately instructed this multistep iNKT cell response (67). Interestingly, perturbations at a distant organ were also able to remotely modulate the behavior of hepatic iNKT cells (68). Stroke-associated brain injury induced arrest of iNKT cell crawling in the liver. These cells then started sending out pseudopods to scan around their cell bodies, showing a “pirouetting” behavior that was not observed during cytokine or antigenic stimulations but was mediated by neurotransmitters. This unique behavior of iNKT cells was associated with their IL-10 production upon innervation (68). It is thus inferred from these studies that the multifaceted function of hepatic iNKT cells may be determined by their cellular behaviors.

### Liver-Resident NK Cell—More Than Killers With the Need for Visualization

NK cell represents another liver-enriched lymphocyte population, accounting for up to 50% of total intrahepatic lymphocytes in humans and 10% of that in mice (69). Flow cytometric analysis of mouse liver revealed two phenotypically distinct populations of NK cells, based on their mutually exclusive expression of DX5 and CD49a (70). The DX5^+^CD49a^–^ subsets appeared as circulating conventional NK (cNK) cells that were transiently passing through the liver. On the contrary, the DX5^–^CD49a^+^ subset showed unique features that defined them as a liver-resident NK (LrNK) cell population (70). Importantly, LrNK cells are developmentally separated from cNK cells. They originated from hematopoietic progenitor cells that persistently seed in the adult liver but not from bone marrow where the cNK cells arise (70, 71). Transcriptional factors that instruct LrNK cell development and maintenance include T-box expressed in T cells (T-bet) (71, 72), Hobit (64), and aryl hydrocarbon receptor (AhR) (73).

As an emergent liver-resident innate immune cell population, LrNK cells serve multiple tissue-specific functions. They were shown to confer T- and B-cell-independent innate memory responses (74). In hapten-induced contact hypersensitivity, LrNK but not cNK cells was sufficient to elicit a recall response upon hapten rechallenge (70). LrNK cell-mediated memory response was also observed during viral infections and was dependent on CXCR6, a chemokine receptor that was highly expressed by LrNK but absent on cNK cells (75, 76). LrNK cells expressed an array of immune regulatory molecules. They significantly inhibited the antiviral T cell responses during acute and chronic lymphocytic choriomeningitis virus (LCMV) infections (77) and suppressed the proliferation of autoimmune CD4^+^T cells in cholangitis (78), highlighting a role of LrNK cells in maintaining liver tolerance. LrNK cell-derived IFN-γ, in addition to exerting cytotoxicity against viral infected cells (79), showed hepatoprotective function by upregulating antiapoptotic signals during acute liver injury (80), although the LrNK cells in these two studies were defined as liver innate lymphoid cell 1 (ILC1), a cell category that embodied LrNK and others (81).

While much can be learned from these studies, there is a paucity of description regarding the subtissular localization and dynamic behaviors of LrNK cells. Intravital imaging of LrNK cells is promising in addressing these questions although challenges remain, largely due to their infrequency in mice and the lack of an LrNK cell-specific fluorescent reporter mouse. Nevertheless, by exploiting the cellular dynamics of LrNK cells *in vivo*, our knowledge about their function and ontogeny will be greatly expanded (Figure 2). For example, it would be interesting to image how LrNK cells are primed in an antigen-specific way and how memory LrNK cells are mobilized to a peripheral organ where the cognate antigen is re-encountered. Furthermore, it was recently reported that CD8^+^T cells drove LrNK cell maturation in a cell-contact-dependent manner (82). Dynamic imaging of cellular interactions between these two cell types would provide key insights into the “LrNK cell niche” that is indispensable for their development and education.

## Intravital Imaging Unravels the Mysteries of Intrahepatic T Cell Responses

The liver has been historically considered as an immune privilege organ favoring immune tolerance induction (83). Liver-induced immune tolerance was considered as a major reason for viral persistence during chronic hepatitis B and C virus (HBV and HCV) infection (5, 7). Nonetheless, the liver was also shown as fully competent in mounting robust T cell responses particularly in acute infections (84). This is not a paradox but rather reflecting the complex outcomes of a T cell response in the liver. It is becoming clearer now that the nature of intrahepatic T cell response can be shaped by various factors, including but not limited to the route of antigen exposure, antigen load (85), type of APCs (86), the extent of inflammation (87), and cytokine milieu (88). In this section, we will discuss some key findings that were uncovered by IVM, which shed new light in T cell priming and surveillance in the liver.

### T Cell Priming in the Liver—How Does a Naive T Cell Become Activated in the Liver?

**FIGURE 3:**
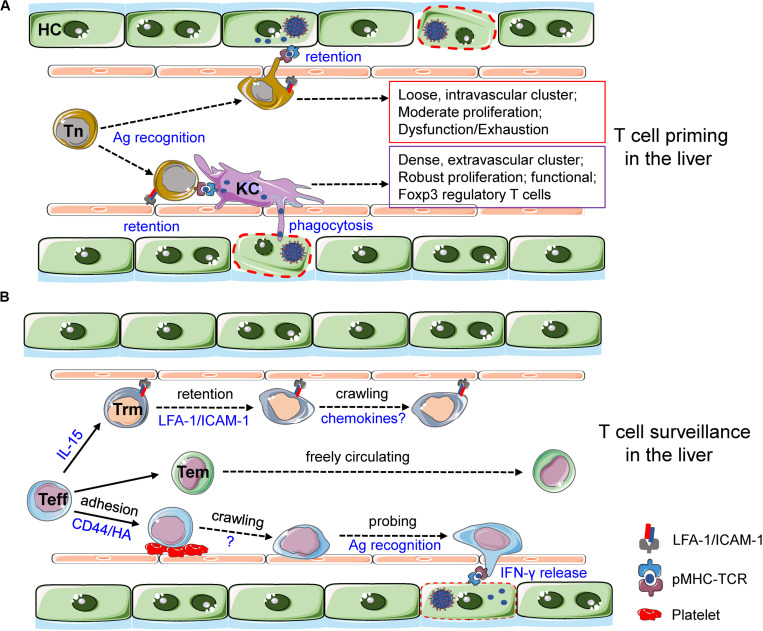
T cell priming and surveillance in the liver. **(A)** Naive T (Tn) cells extend cell protrusions across the fenestrated liver sinusoids to reach infected hepatocytes and sense cognate antigens. Antigen recognition in the absence of inflammation leads to the formation of loose, intravascular T cell clusters. These T cells show moderate proliferative capacity and are dysfunctional. Prolonged antigen stimulation by hepatocytes induces T cell exhaustion. By contrast, KCs phagocytose dead hepatocytes and cross-present cognate antigens, leading to the formation of dense, extravascular T cell clusters. T cells primed by KCs show robust proliferation and are functionally competent. Furthermore, CD4^+^T cell primed by KCs generates regulatory T cells in the absence of inflammation. LFA-1 and ICAM-1-mediated hepatic adhesion of naive T cells is required for T cell priming in the liver. **(B)** Effector T (Teff) cells generated in SLOs dock onto adherent platelets and then crawl along the liver sinusoids to probe the parenchymal via fenestrated LSECs. Once cognate antigens are recognized, Teff cells quickly release IFN-γ to eradicate infections. Some of these effector T cells are retained in the liver vasculature through LFA-1-ICAM-1-mediated adhesion. They differentiate into tissue-resident memory T cells in an IL-15-dependent manner. Liver Trm cells then crawl along the sinusoids via as-yet-unidentified molecular mechanisms where chemokines may play a role. Some other effector T cells differentiate into Tem cells and are freely circulating in the blood.

With the presence of fenestration and the absence of basal membranes, the highly permeable liver sinusoids not only enable a direct priming of circulating naive T cells by infected hepatocytes but also open the door for other hepatic APCs, e.g., LSEC, KCs, and DCs, to grab antigens from the parenchyma. T cell priming by these very different APCs can result in various outcomes in terms of T cell activation, behavior, and function (Figure 3A). A recent study elegantly elucidated the dynamics of CD8^+^T cells primed by different hepatic APCs (89). When the HBV antigens were strictly expressed by hepatocytes, antigen-specific CD8^+^T cells form loose, long-lasting, and intravascular clusters surrounding the portal tracts. These hepatocyte-primed CD8^+^T cells were transcriptionally and epigenetically different from effector T cells. As a consequence, they were dysfunctional and gradually became exhausted if the antigen persisted (89). By contrast, when the antigens were delivered to KCs, CD8^+^T cells fully differentiated into functional competent effector T cells. They formed dense, temporal, and extravascular clusters scattered throughout liver lobules. The depletion of KCs but not DCs completely abolished this T cell priming effect (89), even though the approach used to deplete DCs also eliminated half of KCs (55), suggesting a pivotal and powerful function of KCs in priming CD8^+^T cells. Importantly, these observations revealed a *bona fide* effect of hepatic APCs in CD8^+^T cell priming, as the role of secondary lymphoid organs (SLOs) was experimentally precluded (89).

The priming of CD4^+^T cell in the liver was also visualized. Latex beads covalently coupled with model antigen ovalbumin (OVA) were injected to induce a selective antigen presentation by KCs (87). OVA-specific CD4^+^T cells (OT-II) reduced their cell motility until being completely arrested by antigen-loaded KCs. The antigen-dependent interactions between KCs and OT-II cells can last for hours, resulting in T cell activation and proliferation. However, these OT-II cells differentiated into Foxp3^+^ regulatory T (Treg) cells over time (87), indicating a tolerogenic role of KCs during CD4^+^T cell priming. Liver inflammation induced by carbon tetrachloride treatment strongly dissociated the contacts between KCs and CD4^+^T cells, leading to impaired induction of Treg cells (87). How inflammation fine tunes the KC-primed T cell response is unclear; one could speculate that KCs harbor an array of inhibitory receptors, e.g., CRIg (25) and Clec4g (90) to safeguard the induction of immune inhibitory cells at steady state. Concordantly, LSECs were shown to induce T cell tolerance and dysfunction in various situations (4, 86, 91).

Not only tissue-resident APCs but also emigrant APCs can prime T cells in the liver. *Plasmodium* infection induced a rapid hepatic influx of CD11c^+^ monocyte-derived cells (92). These cells were seen to ingest parasites from dying hepatocytes as early as 40 h postinfection, when the parasites were initially thought to be restricted in hepatocytes (92). After acquiring antigens, these APCs migrated into the liver-draining lymph nodes, where a protective CD8^+^T cell response was induced as reported elsewhere (93, 94).

Hepatic T cell priming can be drastically changed when the liver lobular structure is disrupted. This often happens in chronic infections and is usually coupled with *de novo* formation of structures that restrain the pathogens by heavily populated immune cells. Mycobacterial granuloma in the liver was primarily composed of KCs and inflammatory macrophages. These immotile cells formed dense macrophage clusters to cover the lesion area of the liver (95). KCs were the only cell type containing pathogens in a granuloma. However, uninfected KCs were also observed in the core of granulomas, with a corresponding reduction in these cells in adjacent areas, implying the migration of KCs into granuloma (95, 96). IVM revealed that cognate T cells displayed reduced cell motility and exhibited sustained contact with antigen-presenting macrophages within granulomas (96, 97). However, this resulted in inefficient T cell activation, as CD4^+^T cells showed a low-level and polarized production of effector cytokines. The intrinsic defect of mycobacterial granuloma in priming T cells was due to the limited antigen availability to T cells, likely because antigen-loaded KCs were outnumbered and shielded by antigen-unloaded macrophages (97). A similar structure was reported during chronic viral infections in the liver, namely, intrahepatic myeloid-cell aggregates for T cell population expansion (iMATEs) (98). The iMATEs were mainly constituted by CD11b^+^ monocytes and monocyte-derived inflammatory DCs. In contrast to mycobacterial granuloma, the iMATEs were highly efficient in T cell priming, thereby representing the major venue of a chronically infected liver to drive robust antiviral CD8^+^T cell proliferation (98).

### T Cell Surveillance in the Liver—How Does a T Cell Find Its Target in the Liver?

In most cases of liver infections, effector T cells can be differentiated in the SLOs, from where they migrate into the liver to destroy infected cells (Figure 3B). Using exquisite imaging methods, Iannacone’s group depicted an unappreciated intravascular immunosurveillance program of effector T cells in the liver (99); activated HBV-specific CD8^+^T cells rapidly adhered in the liver independent of integrins and chemokines. Alternatively, attached platelets in liver sinusoids acted as the primary docking sites for these effector T cells (99). CD44–hyaluronan interaction that was critical for hepatic sequestration of leukocytes (100) was involved in platelet adherence (99, 101). After initial binding to platelets, effector T cells started crawling along the liver sinusoids. They concurrently extended cell protrusions traversing the fenestrated endothelium to scan subsinusoidal space. Once reaching an HBV-expressing hepatocyte, the effector T cells were quickly arrested to produce IFN-γ and ultimately extravasated into the parenchyma (99). Noteworthily, interacting with hepatocyte through fenestrations was also observed in naive T cells upon antigen recognition (102). This led to T cell retention via LFA-1/ICAM-1 mediated adhesion (103). The expression of major histocompatibility complex class I (MHC-I) and ICAM-I was not evenly distributed on hepatocytes but was polarized to their perisinusoidal membrane, maximizing the ability of hepatocytes to retain circulating T cells. Hepatic retention of T cells resulted in immune tolerance in the absence of inflammation (103–105). However, a recent study showed that intrahepatic Treg cells were preferentially detained and engulfed by hepatocytes as compared to non-Treg T cells. This phenomenon, termed as enclysis, may represent a novel immunomodulating function for hepatocytes to overcome liver tolerance by deleting Treg cells (106).

Effector memory T (Tem) cells and tissue-resident memory T (Trm) cells exhibited distinct migration patterns in the liver. CD8^+^ Tem cells were rounded and moved freely in blood vessels with occasional and transient interactions with LSECs (107). In sharp contrast, CD8^+^ Trm cells showed an amoeboid shape with high polarity; they crawled along liver sinusoids with a migration speed much slower than that of Tem cells (107). This patrolling behavior of Trm cells could be an ideal pattern to survey reinfected hepatocytes. Interleukin-15 (IL-15), but not cognate antigens in the liver, was pivotal for hepatic Trm cell differentiation (108). Liver CD8^+^ Trm cells do not express CD103, which is a key integrin for Trm to establish tissue residency in many other organs, but alternatively, LFA-1-ICAM-1 interaction was essential for Trm cells to reside in the liver (109).

Intravascular crawling endows T cells with the ability to scan their targets, but how do T cells behave to destroy these targets after finding them? Activated CD8^+^T cells were seen to swarm toward parasites, forming large clusters with up to 25 antigen-specific CD8^+^T cells surrounding one infected hepatocyte (110). Comparing to the “move and search” behavior during intravascular patrolling (99), the swarming behavior of effector T cells may represent a “marshal, break-in and destroy” mission to eliminate infected cells. Indeed, prolonged interaction of CD8^+^T cells with hepatocytes was correlated with apparent death of parasites. Various death phenotypes of parasites were identified by imaging, implying different killing mechanisms by T cells (110), either by direct cytotoxicity or by cytokines (111, 112).

The spatiotemporal features of CD4^+^ effector T cells in the liver are much less understood. Imaging the trafficking of *in vitro* polarized Th1 and Th2 cells revealed a vigorous adhesion of these CD4^+^T cells in inflamed liver sinusoids and postsinusoidal venules. Hepatic adhesion of Th1 and Th2 cells was guided by distinct molecular cues, involving α4β1-integrin and vascular adhesion protein 1 (VAP-1), respectively (113). Since CD4^+^T cell subsets have been discovered with remarkable plasticity (114), whether and how the functional transition of CD4^+^T cells is adapted to their cellular behaviors remains an open question.

## Application of Intravital Imaging in Studying Liver Diseases

With the many advantages of IVM in recoding the wild lives of immune cells (16), it has been widely used to investigate the immunopathogenesis of various liver diseases, ranging from infection, inflammation, to cancer. Immune cell dynamics during liver infection and acute liver injury have been extensively studied in the past decade and has been reviewed elsewhere (1, 17). We will briefly outline some of these researches that we have not mentioned in previous sections. We will also discuss the current applications of intravital imaging in studying chronic liver diseases including cancers.

### Liver Infections

The liver is the major organ in clearing bloodstream bacterial infections. Circulating methicillin-resistant *Staphylococcus aureus* (MRSA) were quickly captured by KCs, culminating in up to 90% of the total inoculum sequestered inside KCs (22). The majority of captured bacteria was then killed in the phagolysosome by reactive oxygen species, but a small part of them survived and replicated intracellularly (115). Hence, KCs can act as a shelter for surviving bacteria to avoid elimination by host immunity. This was reminiscent of intracellular bacterial infections, such as *Listeria monocytogenes* (LM), which eventually killed the host KCs through listeriolysin O-induced necroptosis (49). Similarly, some MRSA managed to lyse KCs and translocated into the peritoneum, a place where they were phagocytosed by GATA-binding factor 6 (Gata6^+^) cavity macrophages and then shuttered to the kidneys to establish renal infection (116). Platelets were quickly docked onto the surface of KCs, forming large platelet aggregates that encase macrophages to prevent attached bacteria from escaping (117). A recent report suggested a role of staphylococcal α-toxin in initiating platelet nucleation on KCs. Persistent release of α-toxin thereafter from intracellular MRSA exacerbated the platelet aggregation, resulting in intravascular thrombosis and tissue injury (118).

The intracellular localization prevented bacteria from killing by neutrophils (115). As such, neutrophils were dispensable for the early control of bloodstream bacterial infections (119). Alternatively, neutrophils produced neutrophil extracellular traps (NETs) during infections (120, 121). These DNA-composed sticky structures were decorated with histones and proteinases, endowing them with the ability to ensnare and kill bacteria in the liver sinusoids (120). Thereby, NETs can consolidate the immune barrier function of liver by diminishing the dissemination of bacteria, though usually at a cost of causing collateral tissue damages (121, 122). Taking advantage of IVM in studying the very dynamic response of platelets *in vivo*, NETs were found to induce intravascular coagulation, which exacerbate tissue damages during sepsis (122, 123).

Unlike bacteria, parasites were not immediately caught by KCs. A part of circulating *Plasmodium* sporozoites were seen to abruptly adhere to the liver endothelium and glided along the sinusoids toward a KC. Instead of being phagocytosed, these parasites penetrated the KC and then squeezed into the parenchyma, where they traversed several hepatocytes before finally invading one (124). Since parasite-crossing permanently damaged the membrane integrity of the traversed cells, KC traversal maybe an immune evasion strategy for parasites to survive during liver-stage infection (125). How blood-borne hepatotropic virus, such as HBV and HCV, cross the liver vessels and establish infections in hepatocytes remains unknown; imaging this process *in vivo* could provide important implications for blocking viral transmission. As a clue, it was shown that visualizing and tracking of tiny viral particles in the bloodstream was feasible, which revealed a preferential uptake of oncolytic virus by KCs over other liver cell populations (126). Noteworthily, current studies are mostly focused on infections that are induced by a single type of pathogen. Polymicrobial infections are on the rise but often neglected, in which the disease outcome can be dramatically complicated and exacerbated by simultaneous or sequential infection with two or more different microorganisms (127). It would be intriguing to image these different microbes during a polymicrobial infection to see how they affect the colonization and clearance of each other *in vivo*.

### Acute Liver Injury

The hepatic immune responses to sterile injury are thoroughly characterized by IVM using a focal liver injury model. Burning a tiny area of the liver surface resulted in necrotic cell death and swarming of neutrophils toward the lesion. This directional neutrophil movement was guided by a coordinated effect of many chemoattractants and intracellular signaling molecules (66), including DAMPs, chemokines, and Btk signalosomes (128). The recruited neutrophils performed a critical tissue-repairing task by accelerating angiogenesis (129). Platelets rapidly accumulated at the edge of the lesion and facilitated the entry of neutrophils into the injured tissue (130). CCR2^+^ inflammatory monocytes arrived a little later than neutrophils, but they stopped migrating at the boundary to encompass the lesion (131). iNKT cell-derived IL-4 at this stage instructed a functional transition of monocytes from inflammatory to reparative (67). Reparative monocytes gradually lost CCR2 but obtained CX3CR1 expression, with a concurrently increased ability to infiltrate into the core of injury to ensure proper wound healing (131). Peritoneal macrophages were shown to traverse the liver mesothelium and cover onto the lesion area, facilitating the tissue repair by dismantling DNA from necrotic cells (132).

The cell dynamics and functions of inflammatory cells unraveled using the burn injury model have been extensively confirmed in more clinical-relevant acute liver injury models. During acetaminophen overdose-induced hepatotoxicity, CCR2^+^ monocytes were arrested by necrotic hepatocytes to form dense clusters around the lesion areas. These cells were proinflammatory and aggravated liver injury at the early stage until a functional transition toward anti-inflammatory occurred (133). Neutrophils crawled to the heavily DNA-deposited necrotic area and patrolled inside the lesion (134), exhibiting behaviors that resembled what was found in the burn injury model. Neutrophil recruitment in both models depended on chemoattractants such as *N*-formyl-methionyl-leucyl-phenylalanine (fMLP) and CXCL2 (134), but the signaling pathways in initiating and amplifying neutrophil infiltration were divergent (135). Similar migration patterns of neutrophils were also reported during hepatic ischemia–reperfusion-induced liver injury (136, 137). The timely clearance of recruited neutrophils and monocytes from the injured site is crucial for tissue repair. Although this is generally considered as a result of macrophage efferocytosis, it was not apparently seen *in vivo* (129). Further studies are required to record the fate of these presumably short-lived cells during the resolution of inflammation, despite the possibility of reverse transmigration was proposed (129).

### Chronic Liver Diseases

We are at the beginning of using IVM to study chronic liver diseases. A major obstacle for intravital imaging of a chronically inflamed liver is the strong autofluorescence that overrides the fluorescent signal of labeled immune cells (138). By optimizing optical modules to minimize autofluorescence, visualization of the immune cell dynamics in non-alcoholic fatty liver diseases (NFALDs) was recently achieved (138). By exploiting this new method and the well-established abdominal imaging window, *in vivo* imaging the chronic inflammatory responses that contribute to the transformation from NFALD to non-alcoholic steatohepatitis (NASH) is possible and could be promising for early diagnosis and prevention. Interestingly, platelet recruitment to the fatty liver preceded leukocyte infiltration during NFALD. Adherent platelets were primarily attracted by and interacted with KCs to stimulate the releases of proinflammatory cytokines and chemokines, by which platelets promoted the progression of NFALD to NASH (101).

The application of IVM during chronic liver diseases can be expanded beyond characterizing immune cell dynamics. This technique enables direct visualization of blood and bile flow in living animals, both of which are notoriously difficult to measure *in vitro*, thereby becoming a powerful tool to evaluate the blood–bile barrier (BBIB) integrity. Blood flow in the sinusoids can be routinely monitored using fluorophore-labeled dextran (139). Bile flow can be visualized by injection of 6-carboxyfluorescein diacetate, a probe that is selectively taken up by hepatocytes, hydrolyzed to fluorescent carboxyfluorescein (green fluorescence), and secreted into the biliary canaliculi as early as 2 min after injection (140). With this method, the BBIB integrity has been elegantly examined in multiple settings of chronic liver diseases, especially during cholestasis (141–143).

### Liver Cancer

IVM has been well exploited in studying liver metastasis (144, 145). Circulating tumor cells can easily traverse the highly permeable liver sinusoids to establish colonization, making the liver as a metastasis-prone organ. A large part of circulating tumor cells were seen to quickly adhere to the liver sinusoids without being efficiently ingested by KCs (146). The molecular mechanism for these cancer cells to evade KC phagocytosis remained to be identified; lack of efficient opsonization may be a clue (42). Neutrophils fostered liver metastasis by different mechanisms. They acted as a docking site for cancer cells to adhere in liver sinusoids before breaching the parenchymal. NETs induction either by primary tumors (147) or infections (148) can further enhance the intravascular arrest and seeding of metastatic cancer cells. An abdominal imaging window was developed for long-term visualization of liver metastasis over 14 days (149). It revealed an unappreciated premicrometastasis stage that was resulted from a massive proliferation of a single extravasated tumor cell. Tumor cells at this stage were highly mobile and proliferative and avoided interacting with each other, but they lost these characteristics as the metastatic tumor grew (149). In most cases, liver intravital imaging is an end-point experiment with a short time window for observation, making it difficult to study primary liver cancer that usually takes at least months to occur in mouse models. The abdominal imaging window will offer a great opportunity to interrogate the immune cell dynamics during the initiation and progression of primary liver cancers. Moreover, taking advantage of IVM as an important tool to visualize the *in vivo* distribution of drugs (150) and adoptively transferred cells (15), it could provide valuable information for optimizing cancer immunotherapy against liver cancers, such as immune checkpoint blockade and adoptive cell therapy.

## Concluding Remarks

Intravital imaging opens a new window in the area of liver immunology. By examining the spatial organization, dynamic behavior, and cellular interactions of liver immune cells, great advances have been made in unraveling the function of liver-resident innate immune cells and in dissecting the kinetics of hepatic T cell responses, as discussed above. However, many questions remained to be explored by this cutting-edge technique. Some key liver cell populations demand for visualization, not only including immune cells that are abundant in the liver, such as B cells, NK cells, and γδT cells, but also for non-immune liver-resident cells, such as HSCs. The space of Disse is inhabited by HSCs at a density much greater than previously thought (20). Inspired by observations that these cells intimately contacted with monocytes and KCs (20), dynamically visualizing HSC and immune cell interactions during liver diseases has warranted further investigation. Moreover, multireporter systems need to be developed for better characterizing cellular crosstalk in the liver. Liver sinusoids are crowded with many cell types during infection and inflammation. An immune cell may have to simultaneously or sequentially contact with multiple cell types in such a narrow space to exert a proper function. Characterizing these cellular interactions may be fundamental for therapeutically targeting immune cells in liver diseases. Lastly, unprecedented methods can be created by integrating IVM with other techniques to fulfill a special research purpose. For instance, the “NICHE-seq” (151), which combines photoactivable reporters, single-cell sequencing, and IVM, can be useful to illustrate the spatial heterogeneity of liver cells, a phenomenon well-known as liver zonation (152). With an in-depth understanding of liver immune response by *in vivo* imaging, our chance to conquer liver disease will be improved.

## Author Contributions

LL and ZZ wrote the manuscript. ZZ conceived and supervised the study. Both authors contributed to the article and approved the submitted version.

## Conflict of Interest

The author declares that the research was conducted in the absence of any commercial or financial relationships that could be construed as a potential conflict of interest. The reviewer BS declared a past co-authorship with one of the authors ZZ to the handling editor.

## Abbreviations

α -Galcer: α -galactosylceramide
AhR: aryl hydrocarbon receptor
APC: antigen-presenting cell
BBIB: blood–bile barrier
Blimp1: B lymphocyte-induced maturation protein 1
C3: complement component 3
CD: cluster of differentiation
CLEC4f: C-type lectin domain family 4 member f
cNK: conventional natural killer cell
CRIg: complement receptor of the Ig superfamily
CSF1-R: colony-stimulating factor 1 receptor
CX_3_CR1: C-X 3-C motif chemokine receptor 1
CXCL: C-X -C motif chemokine ligand
CXCR: C-X -C motif chemokine receptor
DC: dendritic cell
EPEC: enteropathogenic *Escherichia coli*
Gata6: GATA-binding factor 6
GFP: green fluorescent protein
HBV: hepatitis B virus
HCV: hepatitis C virus
Hobit: homolog of Blimp1 in T cells
HSC: hepatic stellate cell
IC: immune complex
ICAM-1: intercellular adhesion molecule 1
IFN: interferon
IgM: immunoglobulin M
IL: interleukin
ILC: innate lymphoid cell
iMATEs: intrahepatic myeloid-cell aggregates for T cell population expansion
iNKT: invariant natural killer T cell
IVM: intravital microscopy
KC: Kupffer cell
LCMV: lymphocytic choriomeningitis virus
LFA-1: lymphocyte-function-associated antigen 1
LM: *Listeria monocytogenes*
LrNK: liver-resident natural killer cell
LSECs: liver sinusoidal endothelial cell
MHC I: major histocompatibility complex class I
MRSA: methicillin-resistant *Staphylococcus aureus*
NASH: non-alcoholic steatohepatitis
NETs: neutrophil extracellular traps
NFALD: non-alcoholic fatty liver diseases
NK: natural killer cells
OVA: ovalbumin
PLZF: promyelocytic leukemia zinc finger
SLO: secondary lymphoid organ
T-bet: T-box expressed in T cell
Tem: effector memory T cell
Tim-4: T cell membrane protein 4
Treg: regulatory T cell
Trm: tissue-resident memory T cell
VAP-1: vascular adhesion protein 1.
